# Antimicrobial Diterpenoids of *Wedelia trilobata* (L.) Hitchc

**DOI:** 10.3390/molecules21040457

**Published:** 2016-04-07

**Authors:** Shi-Fei Li, Jia-Yin Ding, Ya-Ting Li, Xiao-Jiang Hao, Shun-Lin Li

**Affiliations:** 1 State Key Laboratory of Phytochemistry and Plant Resources in West China, Kunming Institute of Botany, Chinese Academy of Sciences, Kunming 650201, China; lisf@sxu.edu.cn (S.-F.L.); djyjolly@163.com (J.-Y.D.); 2 Institute of Molecular Science, Shanxi University, Taiyuan 030006, China; 3 Shanxi Zhendong Pharmaceutical Co., Ltd., Changzhi 047100, China; sarahlyt@163.com

**Keywords:** invasive weed, *Wedelia trilobata*, *ent*-kaurane diterpenoids, antimicrobial

## Abstract

Continued interest in the metabolites of *Wedelia trilobata* (L.) Hitchc, a notoriously invasive weed in South China, led to the isolation of twenty-six *ent*-kaurane diterpenoids, including seven new ones **1**–**7**. Their structures and relative configuration were elucidated on the basis of extensive spectroscopic analysis, including 1D- and 2D-NMR experiments. The antimicrobial activities of all isolated diterpenoids were evaluated against a panel of bacteria and fungi.

## 1. Introduction

**Figure 1:**
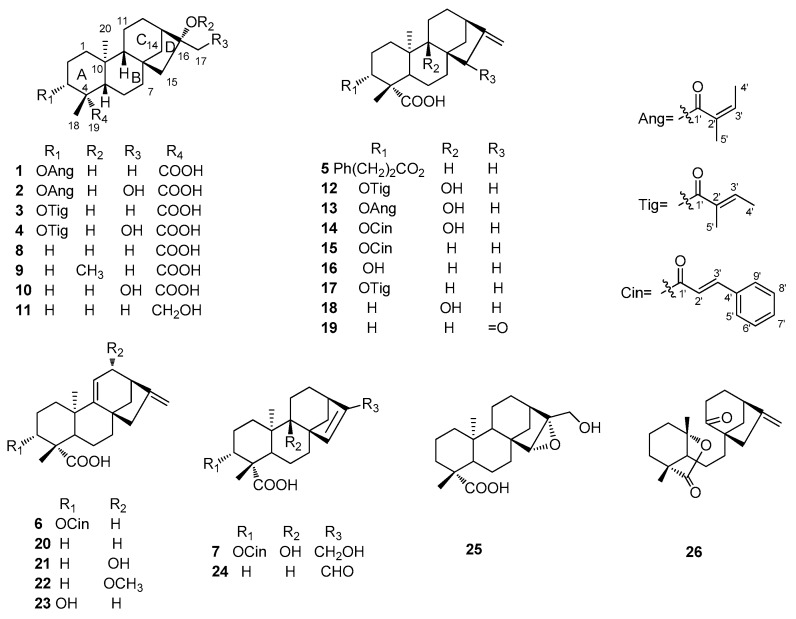
Chemical structures of **1**–**26** from *Wedelia trilobata*.

*Wedelia trilobata* is a notoriously invasive weed in a wide range of tropical and subtropical areas [1]. In southern China, this creeping, matforming perennial herb has caused significant damage to farmlands, forests, and orchards [2,3]. Studies have shown that *W. trilobata* has a strong allelopathic potential on neighboring native plants [4,5]. The major chemical constituents of *W.* *trilobata* are *ent*-kaurane diterpenes, sesquiterpene lactones, and triterpenes with a variety of biological activities, such as antibacterial, antitumor, hepatoprotective, and central nervous system depressant properties [6]. We previously reported ten eudesmanolides isolated from this plant as potential inducers of plant systemic acquired resistance [7]. As continuation of that work, twenty-six *ent*-kaurane diterpenoids including seven new ones **1**–**7** were obtained from the whole plant *W. trilobata* (Figure 1). All diterpenoids were evaluated against a panel of bacteria and fungi, and compounds **2**, **4**, **7**, **10**, **12**, and **13** showed weak inhibitory activities against *Monilia albicans* with MICs of *ca.* 125 μg/mL. Herein, we report the isolation and structural elucidation of these compounds, as well as their antimicrobial properties.

## 2. Results and Discussion

### 2.1. Structure Elucidation of Compounds

Compound **1** was obtained as a white amorphous powder, with a molecular formula determined as C_25_H_38_O_5_ on the basis of HREIMS which indicated a molecular ion peak at *m*/*z* 418.2722 M^+^ (calcd. for C_25_H_38_O_5_, 418,2719). The IR spectrum revealed absorption bands of hydroxyl (3431 cm^−1^) and carbonyl (1711 cm^−1^) groups. In the ^1^H-NMR spectrum (Table 1), the downfield olefinic proton at δ_H_ 6.02 (1H, q, *J* = 7.0 Hz) and two methyl signals at δ_H_ 1.82 (3H, s) and 1.92 (3H, d, *J* = 7.0 Hz), indicated the presence of an angeloyloxy group in **1** [8].

**Figure 2:**
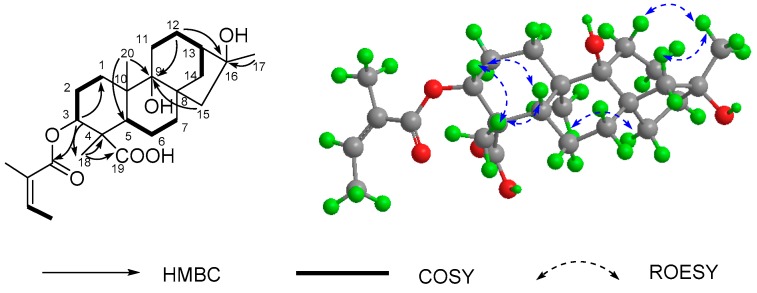
Key 2D-NMR data of **1**.

Apart from five carbon signals assigned to the angeloyloxy group (δ_C_ 167.9, 128.1, 138.6, 20.9, and 16.0), the ^13^C-NMR (DEPT) spectrum of **1** (Table 1) also exhibited 20 carbons composed of three methyls, eight methylenes, four methines (one oxygenated), and five quaternary carbons, which were consistent with a skeleton of an *ent*-kauranoid [9]. In particular, the NMR spectroscopic features of **1** are similar to those of **8** (16α-hydroxy-*ent*-kauran-19-oic acid), and only differed in the appearance of an angeloyloxy group at C-3 in **1**. It was also confirmed by the chemical shift value of C-3 (δ_C_ 78.9, CH), C-9 (δ_C_ 56.1, CH) and the HMBC correlations (Figure 2) from H-3 (δ_H_ 4.50, dd, *J* = 12.2, 4.7 Hz) to C-1′ (δ_C_ 167.9, C), C-1 (δ_C_ 38.9, CH_2_), and C-18 (δ_C_ 24.7, CH_3_) as well as the correlations from Me-20, H-12, and H-15 to C-9, and from the methyl at C-4 (Me-18) to a downfield quaternary carbon (C-19) at δ_C_ 178.1. The ROESY correlations of H-3 with H-5 and H_3_-18 suggested that the angeloyloxy was α-orientated, and the hydroxy at C-16 was also assigned as α-orientated by the ROESY correlations of H_3_-17 with H_2_-11 and H-14β along with the ROESY correlations of H_3_-20 with H_2_-15. Consequently, the structure of **1** was finally determined as 3α-angeloyloxy-16α-hydroxy-*ent*-kauran-19-oic acid.

Compound **2** had the molecular formula C_25_H_38_O_6_ as determined by the HREIMS, with 16 mass units more than **1**. The ^1^H- and ^13^C-NMR data similarities between **2** and **1** (Table 1 and Table 2) suggested that they were structural analogues. As compared with compound **1**, the main differences were due to the presence of a hydroxymethyl group (δ_C_ 66.8) and the absence of a methyl group in **2**. The hydroxymethyl group was assigned to C-17 by the HMBC correlations of H_2_-17 to C-14, C-15, and C-16. Therefore, the structure of **2** was established as shown.

Compounds **3** and **4** showed the same mass units as those of **1** and **2**, respectively, on the basis of the HREIMS. The 1D NMR data of **3** and **4** (Table 1 and Table 2) also closely resembled those of **1** and **2**, respectively, except for the presence of the tigloyloxy group at C-3 of **3** and **4** instead of the angeloyloxy group. These conclusions were verified by the HMBC correlations from H-3′ (δ_H_ 6.80 in **3**, δ_H_ 6.89 in **4**) and H-3 (δ_H_ 4.50 in **3**, δ_H_ 4.50 in **4**) to C-1′ (δ_C_ 167.9 in **3**, δ_C_ 169.4 in **4**). The NMR data suggested that compounds **3** and **4** possessed the same relative configuration as those of **1** and **2**, respectively. Thus, compounds **3** and **4** were determined as 3α-tigloyloxy-16α-hydroxy-*ent*-kauran-19-oic acid and 3α-tigloyloxy-16α, 17-dihydroxy-*ent*-kauran-19-oic acid, respectively.

Compound **5**, a white powder, possessed the molecular formula C_29_H_38_O_4_, as determined by the HREIMS, ^13^C-NMR (Table 2) and DEPT data. Comparison of the 1D- and 2D-NMR spectroscopic data of **5** with those of 3*α*-cinnamoyloxy-*ent*-kaur-16-en-19-oic acid (**15**) revealed that their structures were closely similar to each other. The only difference between them was that the double bond of the cinnamoyloxy group at C-3 in **15** was reduced in **5**, which was supported by the molecular weights of **5**, showing two mass units more than those of **15**. This was further confirmed by the HMBC cross-peaks of H-2′ and H-3′ with C-1′ and C-4′. The α-orientation of the 3-dihydrocinnamoyloxy group was apparent from the ROESY correlations of H-3β with H-5β and H_3_-18β. Thus, compound **5** was determined as 3α-dihydrocinnamoyloxy-*ent*-kaur-16-en-19-oic acid.

The molecular formula of compound **6** was deduced as C_29_H_34_O_4_ on the basis of the positive HREIMS at *m*/*z* 446, 2463 [M]^+^ (calcd. for 446,2457). The ^1^H- and ^13^C-NMR data of **6** (Table 1 and Table 2) showed many similarities to those of **20**, indicating that they were structural analogues as *ent*-kaura-9(11),16-dien-19-oic acid. As compared with compound **20**, the obvious difference was due to the presence of one more cinnamoyloxy group at C-3 in **6**. HMBC correlations from H-3, H-2′, and H-3′ to C-1′ further validated the conclusion above. The ROESY correlations of H-3 with H-5 and H_3_-18 suggested that the cinnamoyloxy was α-orientated. Consequently, the structure of **6** was determined as 3α-cinnamoyloxy-*ent*-kaura-9(11), 16-dien-19-oic acid.

Compound **7** was isolated as a white powder, and its molecular formula was determined as C_29_H_36_O_6_ by HREIMS based on *m*/*z* 480.2508 [M]^+^ (calcd. for 480,2512). The IR spectrum showed absorptions at 3441 (OH), 1701 (C = O), and 1639 and 1449 cm^−1^ (aromatic C = C). The presence of a cinnamoyloxy moiety was deduced by comparison with the NMR data (Table 1 and Table 2) of compound **6**. Besides this cinnamoyloxy moiety, the remaining twenty C-atoms included a trisubstituted double bond (δ_H_ 5.58 (br. s); δ_C_ 134.2 and 149.2), a carboxyl group (δ_C_ 176.0), an O-bearing methylene group (δ_H_ 4.10 (d, *J* = 14.3 Hz) and 4.14 (d, *J* = 14.3 Hz); δ_C_ 60.9), and an O-bearing quaternary carbon (δ_C_ 75.6). Further analyses demonstrated that compound **7** showed a closely similar NMR pattern to that of **6**, indicating that compound **7** was a structural analogue of *ent*-kaurane-19-oic acid. The double bond was located between C-15 and C-16 by the HMBC cross-peaks of H-15 with C-8, C-14, C-16 and C-17. Meanwhile, the O-bearing methylene group was only connected to C-17 by the HMBC correlations from H_2_-17 to C-14, C-15, and C-16. At last, the O-bearing quaternary carbon could be attributed to C-9 due to the HMBC correlations of H_2_-7, H_2_-11, and H_3_-20 to C-9. The relative configuration of **7** was shown to be identical with that of 6 by NMR analysis. Thus, compound **7** was determined as 3α-cinnamoyloxy-9β, 17-dihydroxy-*ent*-kaur-15-en-19-oic acid.

Nineteen known *ent*-kaurane derivatives, namely 16α-hydroxy-*ent*-kauran-19-oic acid (**8**) [10], 16α-methoxy-17-hydroxy-*ent*-kauran-19-oic acid (**9**) [11], 16α-17-dihydroxy-*ent*-kauran-19-oic acid (**10**) [12], 16α, 18-dihydroxy-*ent*-kaurane (**11**) [13], 3α-tigloyloxypterokaurene L3 (**12**) [14], 3α-angeloyloxy-9β-hydroxy-*ent*-kaur-16-en-19-oic acid (**13**) [15], 3α-cinnamoyloxy-9β-hydroxy-*ent*-kaur-16-en-19-oic acid (**14**) [15], 3α-cinnamoyloxy-*ent*-kaur-16-en-19-oic acid (**15**) [12], 3α-hydroxy-*ent*-kaur-16-en-19-oic acid (**16**) [16], 3α-tiglinoyloxy-*ent*-kaur-16-en-19-oic acid *ent*-kaura-9 (**17**) [8], *ent*-9α-hydroxy-16-kauren-19-oic acid (**18**) [17], *ent*-15-oxokaur-16-en-19-oic acid (**19**) [18], (11),16-dien-19-oic acid (**20**) [19], 12α-hydroxy-*ent*-kaur-9(11),16-dien-19-oic acid (**21**) [20], 12α-methoxy-*ent*-kaur-9(11),16-dien-19-oic acid (**22**) [21], 3α-hydroxy-*ent*-kaura-9(11), 16-dien-19-oic acid (**23**) [21], *ent*-17-oxokaur-15-en-19-oic acid (**24**) [22], 15α,16α-epoxy-17-hydroxy-*ent*-kauran-19-oic acid (**25**) [12], and wedeliaseccokaurenolide (seco) (**26**) [14], were also isolated. Their structures were identified on the basis of spectroscopic analysis and comparison with reported data.

The *in vitro* antimicrobial activities of all *ent*-kaurane derivatives isolated were tested against *Pseudomonas aeruginosa* (ATCC 27853), *Staphyloccocus aureus* (ATCC 25923), *Monilia albicans* (ATCC Y0109) and *Escherichia coli* (ATCC 25922) using an agar well diffusion method [23]. Compounds **2**, **4**, **7**, **10**, **12**, and **13** showed weak activities against *M. albicans* (zone of inhibition > 10 mm at 1 mg/mL). The minimum inhibitory concentrations (MICs) of compounds above against *M. albicans*, 1R (R: methicillin-resistant *M. albicans*), 2R, 3R, 4R, 5R, and 535R were determined by the 2-fold dilution method (Table 3). Fluconazole was used as standard drug for comparison.

### 2.2. Evaluation of Anti-Micobial Activity

**Figure 3:**
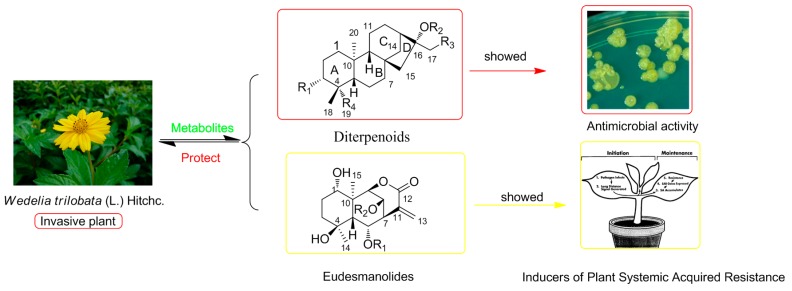
The correlations between *Wedelia trilobata* and its metabolites.

In summary, seven new and nineteen known *ent*-kaurane diterpenoid metabolites were obtained from whole plant *W. trilobata*, and some compounds exhibited weak antimicrobial activities. Moreover, we previously reported ten eudesmanolides as potential inducers of plant systemic acquired resistance isolated from this species [7]. Above all, a conclusion that can be drawn is that diterpenes and sesquiterpenes are the main metabolites of *W. trilobata* and they may be significant as chemical defenses allowing this notoriously invasive weed to adapt to varying surroundings rapidly and effectively (Figure 3).

## 3. Experimental Section

### 3.1. General Procedures

1D- and 2D-NMR spectra were recorder on either an AM-400 or a DRX-500 or an Avance III-600 spectrometer (Bruker, Karlsruhe, Germany) with TMS as an internal standard. Unless otherwise specified, chemical shifts (δ) were expressed in ppm. MS were measured on a HPLC-Thermo Finnigan LCQ Advantage ion trap mass spectrometer (Waters, Milford, PA, USA). Optical rotation was determined on a SEPA-300 polarimeter (Horiba, Tokyo, Japan). UV spectroscopic data were measured on a 210A double-beam spectrophotometer (Shimadzu, Kyoto, Japan). IR spectra of samples in KBr discs were recorded on a Tensor-27 spectrometer with KBr pellets (Bruker, Rheinstetten, Germany). Column chromatography (CC) was carried out on silica gel G (100−200 mesh, 200−300 mesh, Qingdao Haiyang Chemical Co., Qingdao, China), silica gel H (10−40 μm, Qingdao Haiyang Chemical Co.), Sephadex LH-20 (40−70 μm, Amersham Pharmacia Biotech AB, Uppsala, Sweden), and Lichroprep RP-18 gel (20−45 μm, Merck, Darmstadt, Germany). Semi-preparative HPLC was performed on an Agilent 1200 series instrument equipped with a quaternary pump, a vacuum degasser, an auto-sampler, a thermos-tatted column compartment with a Zorbax SB-C18 (10 μm; Agilent Co. Ltd, St. Louis, MO, USA) column (i.d. 9.4 mm × 250 mm), and a diode array detector. Thin-layer chromatography (TLC) was conducted on precoated silica gel plates GF 254 (Qingdao Haiyang Chemical Co.). TLC spots were visualized by heating silica gel plates sprayed with 10% H_2_SO_4_ in EtOH.

### 3.2. Plant Material

The whole plant of *Wedelia trilobata* (L.) Hitchc was collected in Simao, Yunnan Province, China, in August 2011. The specimen was identified by Yu Chen of Kunming Institute of Botany (KIB), Chinese Academy of Sciences (CAS). A voucher specimen (H20110805) has been deposited in the State Key Laboratory of Phytochemistry and Plant Resources in West China, Kunming Institute of Botany.

### 3.3. Extraction and Isolation

Dried powder of the whole plant of *W. trilobata* (9 kg) was extracted under reflux with MeOH (70 L, three times for 4, 4, and 3 h). The solvent was removed under reduced pressure to give a residue (1020.0 g, 11.3%), which was suspended with water and then extracted with petroleum ether, chloroform, EtOAc, and *n*-BuOH successively. The extracts were evaporated under vacuum to afford the corresponding extracts of petroleum ether (200.0 g), chloroform (90.0 g), EtOAc (90.0 g), and *n*-BuOH (380.0 g). The EtOAc (90.0 g) extract was separated with a silica gel G column (100−200 mesh, 10 cm × 120 cm, 450.0 g), eluted with petroleum ether/acetone (*v*/*v* = 9:1, 7:3, 6:4, 1:1, 0:1, each 10 L), to give five fractions (1−5). Fraction 3 (12.0 g) was extensively chromatographed over column of silica gel (3.6 × 100 cm, 36.0 g) and Sephadex LH-20 (CHCl_3_−MeOH, 1:1, 3.2 × 140 cm) to afford compounds **1** (1.2 mg), **2** (10.1 mg), **3** (0.4 mg), **4** (20.0 mg), **11** (2.3 mg), **12** (3.0 mg), **13** (4.5 mg), **14** (4.8 mg) and **15** (4.0 mg). Fraction 4 (7.0 g) was subjected to a column of reversed-phase silica gel (5 cm × 50 cm, 100 g) eluted with a MeOH/H_2_O (50/50 to 100/0) gradient to yield four sub-fractions (A−D). Sub-fraction B (200 mg) was purified by semi-preparative HPLC with 70% MeOH in H_2_O as the mobile phase to yield **5** (6.7 mg, t_R_ = 13.54 min), **6** (1.8 mg, t_R_ = 14.30 min), and **7** (13.0 mg, t_R_ = 15.75 min). Sub-fraction C (2.2 g) was applied to silica gel (5 cm × 80 cm, 30 g) eluted with DCM (dichloromethanemethylene chloride)/MeOH (100/1 to 10/1) to yield four sub-fractions (C1 to C4), respectively. Sub-fraction C2 (320.0 mg) was purified by semi-preparative HPLC with 72% MeOH in H_2_O as the mobile phase to yield **16** (26.8 mg, t_R_ = 18.23 min), **17** (17.2 mg, t_R_ = 20.40 min), and **18** (1.7 mg, t_R_ = 21.62 min), and in a similar procedure, sub-fraction C3 (200.0 mg) yielded **19** (2.3 mg, t_R_ = 16.52 min), **20** (7.0 mg, t_R_ = 17.24 min), **21** (8.0 mg, t_R_ = 19.93 min), and **25** (4.0 mg, t_R_ = 20.92 min). By using the same purification procedures, sub-fraction C4 yielded **22** (6.5 mg), **23** (5.0 mg), and **24** (14.0 mg). Sub-fraction D was subjected to a column of Sephadex LH-20 gel (3.2 cm × 140 cm) eluted with MeOH to obtain six major sub-fractions, each of which was then purified by semi-preparative HPLC with the mobile phase MeOH/H_2_O (65/35) to produce compounds **8** (10.0 mg, t_R_ = 12.44 min), **9** (9.0 mg, t_R_ = 14.93 min), **10** (5.0 mg, t_R_ = 15.45 min), and **26** (37.1 mg, t_R_ = 16.75 min).

### 3.4. Data for ***1**–**7***

*3α-Angeloyloxy-16α-hydroxy-ent-kauran-19-oic acid* (**1**): white powder, ${\left[\alpha \right]}_{D}^{20}$: −57.0 (*c* 0.07, MeOH); UV (MeOH) λ_max_ (log ε) 211.8 (3.6) nm; IR (KBr) ν_max_ 3431, 2927, 2854, 1711, 1626, 1461, 1382, 1235, 1163, 1122, 1044 cm^−1^; positive-ion ESI-MS *m*/*z* 441 [M + Na]^+^, 859 [2M + Na]+, HR-EIMS *m*/*z* 418.2722 M^+^ (calcd. for C_25_H_38_O_5_, 418.2719); ^1^H-NMR (CDCl_3_, 600 MHz) and ^13^C-NMR (CDCl_3_, 150 MHz), see Table 1 and Table 2.

*3α-Angeloyloxy-16α,17-dihydroxy-ent-kauran-19-oic acid* (**2**): white powder, ${\left[\alpha \right]}_{D}^{20}$: −50.37 (*c* 0.09, MeOH); UV (MeOH) *λ*_max_ (log ε) 216 (3.79) nm; IR (KBr) ν_max_ 3432, 2931, 2869, 2853, 1707, 1641, 1454, 1356, 1236, 1021, 988 cm^−1^; ESIMS *m*/*z* 457 [M + Na]^+^; HREIMS *m*/*z* 434.2672 [M]^+^ (calcd. for C_25_H_38_O_6_, 434.2668); ^1^H- and ^13^C-NMR data see Table 1 and Table 2.

*3α-Tigloyloxy-16α-hydroxy-ent-kauran-19-oic acid* (**3**): white powder, ${\left[\alpha \right]}_{D}^{20}$: −42.8 (*c* 0.1, MeOH); UV (MeOH) λ_max_ (log ε) 210.2 (3.6) nm; IR (KBr) ν_max_ 3442, 2927, 2854, 1705, 1624, 1446, 1383, 1122, 1080, 1007 cm^−1^; positive-ion ESI-MS *m*/*z* 441 [M + Na]^+^, 859 [2M + Na]+, HR-EIMS *m*/*z* 418.2723 M^+^ (calcd. for C_25_H_38_O_5_, 418.2719); ^1^H-NMR (CDCl_3_, 600 MHz) and ^13^C-NMR (CDCl_3_, 150 MHz), see Table 1 and Table 2.

*3α-Tigloyloxy-16α,17-dihydroxy-ent-kauran-19-oic acid* (**4**): white powder, ${\left[\alpha \right]}_{D}^{20}$: −41.00 (*c* 0.10, MeOH); UV (MeOH) λ_max_ (log ε) 216 (3.84) nm; IR (KBr) ν_max_ 3431, 2929, 2868, 2851, 1706, 1649, 1464, 1384, 1270, 1151, 1126, 1083, 1053 cm^−1^; ESIMS *m*/*z* 457 [M + Na]^+^; HREIMS *m*/*z* 434.2677 [M]^+^ (calcd. for C_25_H_38_O_6_, 434.2668); ^1^H- and ^13^C-NMR data see Table 1 and Table 2.

*3α-Diydrocinnamoyloxy-ent-kaur-16-en-19-oic acid* (**5**): white powder, ${\left[\alpha \right]}_{D}^{20}$: −64.00 (*c* 0.14, MeOH); UV (MeOH) λ_max_ (log ε) 204 (3.96), 261 (2.57) nm; IR (KBr) ν_max_ 3441, 2927, 2855, 1732, 1703, 1631, 1453, 1181 cm^−1^; ESIMS *m*/*z* 473 [M + Na]^+^; HREIMS *m*/*z* 450.2781 [M]^+^ (calcd. for C_29_H_38_O_4_, 450.2770); ^1^H- and ^13^C-NMR data see Table 1 and Table 2.

*3α-Cinnamoyloxy-ent-kaura-9(11),16-dien-19-oic acid* (**6**): white powder, ${\left[\alpha \right]}_{D}^{20}$: −6.32 (*c* 0.14, MeOH); UV (MeOH) λ_max_ (log ε) 203 (3.74), 277 (3.67) nm; IR (KBr) ν_max_ 3450, 2931, 2866, 1636, 1387, 1173 cm^−1^; ESIMS *m*/*z* 469 [M + Na]^+^; HREIMS *m*/*z* 446.2463 [M]^+^ (calcd. for C_29_H_34_O_4_, 446.2457); ^1^H- and ^13^C-NMR data see Table 1 and Table 2.

*3α-Cinnamoyloxy-9β,17-dihydroxy-ent-kaur-15-en-19-oic acid* (**7**): white powder, ${\left[\alpha \right]}_{D}^{20}$: −55.76 (*c* 0.11, MeOH); UV (MeOH) λ_max_ (log ε) 205 (4.21), 215 (4.20), 277 (4.28) nm; IR (KBr) ν_max_ 3441, 2925, 2863, 1701, 1639, 1449, 1311, 1281, 1204, 1185 cm^−1^; ES^−^–MS *m*/*z* 479 [M − H]^−^; HREIMS *m*/*z* 480.2508 [M]^+^ (calcd. for C_29_H_36_O_6_, 480.2512); ^1^H- and ^13^C-NMR data see Table 1 and Table 2.

### 3.5. Antimicrobial Assays

The strains used in antimicrobial tests were obtained from the Research Center of Natural Medicine, Clinical School of Kunming General Hospital of Chengdu Military Command. The test organisms in this bioassay were the bacteria *Pseudomonas aeruginosa* (ATCC 27853), *Staphyloccocus aureus* (ATCC 25923) and *Escherichia coli* (ATCC 25922) (all grown on MH medium) and the fungus *Monilia albicans* (ATCC Y0109) (grown on Sabauraud′s medium). For the agar plate punch assay [23], all tested compounds were dissolved in DMSO at a concentration of 1 mg/mL. Then, 50 μL of the solution was added onto a well (6 mm in diameter) that had been punched in the appropriate agar growth medium smeared with a suspension of the test organism (1.0 × 10^9^ cfu/mL; cfu = colony forming unit). All active compounds with a diameter of inhibition greater than 10 mm were submitted to minimum inhibitory concentration testing. The MICs of compounds **2**, **4**, **7**, **10**, **12**, and **13** against *M. albicans*, 1R (R: methicillin-resistant *M. albicans*), 2R, 3R, 4R, 5R, and 535R were determined using a 2-fold dilution method [23]. The 2-fold serially diluted compounds in MH broth were dispensed into 96-well microtiter plates (100 μL/well), and then an aliquot of 5 × 10^4^ cfu/mL of bacterial culture was added to each well (100 μL/well) to final concentrations in a range of 1.95−250 μg/mL. After incubating at 37 °C for 18 h, the lowest concentration without any colony growth was recorded as the MIC value. The resulting values were compared with the value for a positive control (fluconazole, range 125–250 μg/mL) under the same conditions.

## 4. Conclusions

A systematic chemical search was performed and resulted in the separation of seven new *ent*-kaurane diterpenoids together with nineteen known *ent*-kaurane derivatives from *Wedelia trilobata* (L.) Hitchc, a notoriously invasive weed in South China. The structures of the new compounds were identified based on detailed spectroscopic analysis and comparison with the published data of analogues. All *ent*-kaurane derivatives isolated were tested against *Pseudomonas aeruginosa*, *Staphyloccocus* *aureus*, *Monilia albicans* and *Escherichia coli*, and compounds **2**, **4**, **7**, **10**, **12**, and **13** showed weak activities against *M. albicans*. Taken together with data from prior research [7], the conclusion that diterpenes and sesquiterpenes are main metabolites of *W. trilobata* can be drawn and they may be significant as chemical defenses allowing this notoriously invasive weed to adapt to varying surroundings rapidly and effectively.

## Figures and Tables

**Table 1:** ^1^H-NMR Data for Compounds **1**–**7**.

NO.	1 ^a^	2 ^b^	3 ^a^	4 ^b^	5 ^a^	6 ^c^	7 ^d^
1a	0.97 (1H, *)	1.05 (1H, s)	0.97 (1H, *)	1.04 (1H, m)	1.03 (1H, d, 9.6)	1.55 (1H, m)	1.56 (1H, m)
1b	1.88 (1H, br d, 13.5)	1.96 (1H, m)	1.87 (1H, br d, 13.7)	1.95 (1H, m)	1.94 (1H, br d)	2.01 (1H, *)	2.18 (1H, td, 13.7, 4.3)
2a	1.72 (1H, m)	1.70 (1H, m)	1.69 (1H, m)	1.65 (1H, m)	1.68 (1H, m)	1.87 (1H, m)	1.71 (1H, m)
2b	2.30 (1H, m)	2.46 (1H, m)	2.27 (1H, m)	2.42 (1H, m)	2.33 (1H, m)	2.55 (1H, m)	2.57 (1H, m)
3	4.50 (1H, dd, 12.2, 4.7)	4.56 (1H, dd, 12.1, 4.6)	4.50 (1H, dd, 12.2, 4.7)	4.50 (1H, dd, 12.1, 4.6)	4.52 (1H, dd, 12.2, 4.6)	4.77 (1H, dd, 12.0, 4.8)	4.61 (1H, dd, 12.5, 4.5)
5	1.04 (1H, br d, 11.9)	1.12 (1H, d, 6.4)	1.04 (1H, br d, 11.9)	1.11 (1H, m)	1.08 (1H, m)	1.85 (1H, m)	1.93 (1H, dd, 12.5, 2.2)
6a	1.61 (1H, *)	1.67 (1H, m)	1.61 (1H, *)	1.68 (1H, m)	1.63 (1H, m)	2.01 (1H, *)	1.66 (1H, m)
6b	1.80 (1H, *)	1.87 (1H, m)	1.79 (1H, *)	1.86 (1H, m)	1.84 (1H, m)	2.01 (1H, *)	1.85 (1H, dd, 13.9, 2.4)
7a	1.37 (1H, *)	1.49 (1H, m)	1.37 (1H, *)	1.47 (1H, dd, 10.3, 3.3)	1.45 (1H, m)	1.56 (1H, m)	1.29 (1H, t, 3.2)
7b	1.59 (1H, *)	1.65 (1H, m)	1.59 (1H, *)	1.64 (1H, m)	1.53 (1H, *)	2.10 (1H, m)	2.11 (1H, m)
9	0.90 (1H, br s)	1.04 (1H, br d)	0.91 (1H, br s)	1.03 (1H, br s)	1.04 (1H, br s)		
11a	1.49 (1H, *)	1.63 (2H, *)	1.49 (1H, *)	1.62 (2H, m)	1.53 (1H, *)	5.28 (1H, s)	1.47 (1H, m)
11b	1.49 (1H, *)		1.49 (1H, *)		1.65 (1H, d, 4.9)		1.62 (1H, m)
12a	1.44 (1H, m)	1.51 (1H, m)	1.43 (1H, m)	1.51 (1H, d, 3.8)	1.47 (1H, m)	2.03 (1H, m)	1.42 (1H, dd, 12.9, 5.3)
12b	1.51 (1H, *)	1.63 (1H, *)	1.50 (1H, *)	1.62 (1H, m)	1.56 (1H, m)	2.44 (1H, m)	2.01 (1H, m)
13	1.77 (1H, *)	2.03 (1H, br s)	1.77 (1H, *)	2.02 (1H, br s)	2.62 (1H, s)	2.80 (1H, s)	2.53 (1H, m)
14a	1.55 (1H, m)	1.63 (1H, *)	1.55 (1H, m)	1.63 (1H, m)	1.12 (1H, m)	1.52 (1H, m)	1.48 (1H, dd, 5.0, 2.0)
14b	1.81 (1H, *)	1.89 (1H, m)	1.80 (1H, *)	1.89 (1H, d, 11.3)	1.91 (1H, d, 11.0)	1.65 (1H, m)	2.24 (1H, d, 10.5)
15a	1.50 (1H, *)	1.41 (1H, d, 14.4)	1.50 (1H, *)	1.40 (1H, d, 14.2)	2.05 (1H, br s)	2.24 (1H, d, 15.6)	5.58 (1H, br s)
15b	1.50 (1H, *)	1.53 (1H, d, 14.4)	1.50 (1H, *)	1.54 (1H, d, 14.2)	2.05 (1H, br s)	2.64 (1H, d, 15.6)	
17a	1.30 (3H, s)	3.60 (1H, d, 11.4)	1.30 (3H, s)	3.60 (1H, d, 11.3)	4.74 (1H, s)	4.84 (1H, s)	4.10 (1H, d, 14.3)
17b		3.70 (1H, d, 11.4)		3.70 (1H, d, 11.3)	4.80 (1H, s)	4.95 (1H, s)	4.14 (1H, d, 14.3)
18	1.22 (3H, s)	1.23 (3H, s)	1.20 (3H, s)	1.20 (3H, s)	1.15 (3H, s)	1.35 (3H, s)	1.26 (3H, s)
20	0.97 (3H, s)	1.07 (3H, s)	0.97 (3H, s)	1.07 (3H, s)	1.01 (3H, s)	1.19 (3H, s)	1.18 (3H, s)
3-ester	6.02 (1H, q, 7.0)	6.11 (1H, dq, 7.0, 1.4)	6.80 (1H, q, 7.1)	6.89 (1H, dq, 7.0, 1.2)	2.68 (2H, dd, 15.0, 7.2)	6.46 (1H, d, 16.2)	6.53 (1H, d, 15.8)
	1.92 (3H, d, 7.0)	1.96 (3H, dd, 7.2, 1.5)	1.72 (3H, d, 7.1)	1.79 (3H, d, 7.2)	2.95 (2H, m)	7.70 (1H, d, 16.2)	7.68 (1H, d, 15.8)
	1.82 (3H, s)	1.86 (3H, s)	1.76 (3H, s)	1.81 (3H, s)	7.26 (2H, m)	7.53 (2H, m)	7.67 (2H, m)
					7.19 (2H, *)	7.40 (2H, *)	7.43 (2H, *)
					7.19 (1H, *)	7.40 (1H, *)	7.43 (1H, *)

^a^ Recorded in CDCl_3_ at 400 MHz; ^b^ Recorded in CD_3_OD at 400 MHz; ^c^ Recorded in CDCl_3_ at 600 MHz; ^d^ Recorded in acetone-*d*_6_ at 400 MHz. * Overlapped.

**Table 2:** ^13^C-NMR Data for Compounds **1**–**7**.

No.	1 ^a^	2 ^b^	3 ^a^	4 ^b^	5 ^a^	6 ^c^	7 ^d^
1	38.9 t	40.0 t	38.9 t	40.0 t	38.7 t	39.5 t	30.7 t
2	24.3 t	25.3 t	24.2 t	25.1 t	25.3 t	25.0 t	24.7 t
3	78.9 d	80.7 t	79.0 d	80.8 t	79.0 d	79.5 d	79.9 d
4	48.0 s	48.8 s	48.1 s	49.0 s	47.8 s	49.6 s	48.3 s
5	56.4 d	57.3 d	56.4 d	57.3 d	56.3 d	46.0 d	49.6 d
6	21.9 t	23.0 t	22.0 t	23.0 t	21.4 t	18.9 t	21.5 t
7	41.9 t	43.0 t	41.9 t	43.0 t	40.9 t	38.1 t	34.8 t
8	45.2 s	45.5 s	45.2 s	45.5 s	43.8 s	42.3 s	54.2 s
9	56.1 d	57.3 d	56.0 d	57.3 d	55.1 d	155.6 s	75.6 s
10	39.5 s	40.5 s	39.5 s	40.5 s	39.3 s	38.4 s	44.4 s
11	18.5 t	19.7 t	18.5 t	19.7 t	18.5 t	115.2 t	26.6 t
12	26.9 t	27.2 t	26.9 t	27.2 t	33.0 t	38.0 t	31.5 t
13	49.0 d	46.2 d	48.9 d	46.2 d	43.7 d	41.2 d	41.1 d
14	37.6 t	38.0 t	37.5 t	38.0 t	39.4 t	44.9 t	45.1 t
15	57.5 t	53.5 t	57.5 t	53.5 t	48.7 t	51.0 d	134.2 d
16	79.6 s	82.8 s	79.7 s	82.8 s	155.0 s	158.2 s	149.2 s
17	24.1 q	66.8 t	24.1 q	66.8 t	103.0 t	106.0 t	60.9 t
18	24.7 q	24.5 q	24.7 q	24.5 q	23.6 q	24.3 q	24.4 q
19	178.1 s	177.9 s	178.3 s	178.1 s	180.0 s	178.1 s	176.0 s
20	15.7 q	16.1 q	15.7 q	16.1 q	15.3 q	23.3 q	17.4 q
3-ester	167.9 s	169.4 s	167.9 s	169.4 s	173.0 s	166.8 s	166.9 s
	128.1 s	129.3 s	128.9 s	129.5 s	36.0 t	118.4 d	119.5 d
	138.6 d	139.0 d	137.8 d	138.8 d	30.9 t	145.3 d	145.2 d
	16.0 q	16.0 q	14.7 q	14.4 q	140.0 s	134.5 s	135.4 s
	20.9 q	20.9 q	12.3 q	12.1 q	128.0 d	128.4 d	129.0 d
					128.0 d	129.1 d	131.1 d
					126.0 d	130.5 d	129.8 d

^a^ Recorded in CDCl_3_ at 100 MHz; ^b^ Recorded in CD_3_OD at 100 MHz; ^c^ Recorded in CDCl_3_ at 150 MHz; ^d^ Recorded in acetone-*d*_6_ at 100 MHz.

**Table 3:** Antimicrobial activities of compounds **2**, **4**, **7**, **10**, **12** and **13**.

Compounds	Antimicrobial Activities (MIC in μg/mL)						
	*M. albicans*	1R ^b^	2R	3R	4R	5R	535R
**2**	125	>250	>250	>250	>250	>250	>250
**4**	125	125	>250	>250	>250	>250	>250
**7**	125	>250	>250	>250	>250	>250	>250
**10**	125	>250	>250	>250	>250	>250	>250
**12**	125	>250	>250	>250	>250	>250	>250
**13**	125	>250	>250	>250	>250	>250	>250
Fluconazole ^a^	125	>250	>250	>250	>250	>250	>250

^a^ Positive control; ^b^ R means methicillin-resistant *M. albicans*.
